# Integrating NLP and Ensemble Learning into Next-Generation Firewalls for Robust Malware Detection in Edge Computing

**DOI:** 10.3390/s26020424

**Published:** 2026-01-09

**Authors:** Ramahlapane Lerato Moila, Mthulisi Velempini

**Affiliations:** Department of Computer Science, University of Limpopo, Polokwane 0727, South Africa; mthulisi.velempini@ul.ac.za

**Keywords:** next-generation firewall, edge computing, natural language processing, ensemble learning, cyber threat detection

## Abstract

**Highlights:**

This study proposes a novel NLP–ensemble model for next-generation firewalls that achieves over 95% accuracy in detecting malware within edge computing environments. By integrating synthetic data generation to address class imbalance effectively, the model significantly improves the detection of malicious network traffic, providing a scalable and intelligent defense layer for resource-constrained systems.

**What are the main findings?**

- Proposed NLP–ensemble model achieves 95% and 98% accuracy on cyber threat and CSE-CIC-IDS2018 datasets, respectively.
- Synthetic data generation effectively mitigates class imbalance, drastically improving the minority class (malicious traffic) detection.

**What is the implication of the main finding?**

- Provides a scalable, intelligent defense layer optimized for resource-constrained edge environments.
- Demonstrates a practical pathway for integrating AI-driven, language-based threat recognition into existing NGFW architectures.

**Abstract:**

As edge computing becomes increasingly central to modern digital infrastructure, it also creates opportunities for sophisticated malware attacks that traditional security systems struggle to address. This study proposes a natural language processing (NLP) framework integrated with ensemble learning into next-generation firewalls (NGFWs) to detect and mitigate malware attacks in edge computing environments. The approach leverages unstructured threat intelligence (e.g., cybersecurity reports, logs) by applying NLP techniques, such as TF-IDF vectorization, to convert textual data into structured insights. This process uncovers hidden patterns and entity relationships within system logs. By combining Random Forest (RF) and Logistic Regression (LR) in a soft voting ensemble, the proposed model achieves 95% accuracy on a cyber threat intelligence dataset augmented with synthetic data to address class imbalance, and 98% accuracy on the CSE-CIC-IDS2018 dataset. The study was validated using ANOVA to assess statistical robustness and confusion matrix analysis, both of which confirmed low error rates. The system enhances detection rates and adaptability, providing a scalable defense layer optimized for resource-constrained, latency-sensitive edge environments.

## 1. Introduction

The rapid growth of edge computing, characterized by distributed nodes that process data closer to the source, offers significant opportunities but also introduces novel security challenges. Among these, sophisticated malware attacks pose a critical threat, demanding more intelligent and adaptive defense mechanisms than traditional approaches can provide [1]. Next-generation firewalls (NGFWs), with advanced capabilities such as deep packet inspection and application awareness, represent a crucial line of defense. However, the increasing sophistication of malware, including natural-language-like commands and control (C2) communications, requires a shift toward more context-aware, predictive security measures [2]. This has prompted growing interest in leveraging artificial intelligence (AI), particularly natural language processing (NLP) and ensemble learning, to enhance NGFWs’ malware detection and mitigation capabilities in edge environments.

NLP techniques can analyze network traffic to identify subtle linguistic patterns indicative of malicious intent in C2 communications, phishing attempts, and even in seemingly benign files [3]. Moreover, ensemble learning, which combines the predictions of multiple machine learning models, improves the robustness and accuracy of malware classification while minimizing false positives and negatives in a resource-constrained edge environment [4]. By integrating NLP for intelligent content analysis with ensemble learning to improve decision-making, NGFWs can evolve into proactive, resilient security gateways capable of mitigating the growing threat of malware attacks in distributed computing environments. The primary contribution of this study is the development and validation of an adapted approach that applies ensemble learning to NLP-based feature-extraction outputs, thereby enhancing malware detection in edge-located NGFWs.

This study addresses the following research questions:

- Can NLP techniques apply to session metadata and security logs effectively to detect malicious patterns that evade traditional rule-based NGFW filtering?
- Does a soft-voting ensemble of Random Forest and Logistic Regression provide a collaborative improvement in accuracy and robustness over individual models for this task?
- Can synthetic data generation effectively mitigate class imbalance in cybersecurity datasets to improve the detection of minority-class (malicious) traffic?
- Is the proposed NLP–ensemble framework computationally viable for deployment in resource-constrained edge computing environments?

These questions inform our methodological choices and evaluation, with RQ1 guiding our NLP feature extraction design, RQ2 motivating our ensemble architecture and ablation study, RQ3 shaping our data preprocessing strategy, and RQ4 guiding our analysis of computational overhead and latency.

The remainder of this paper is organized as follows: Section 2 reviews related work on malware detection and NGFWs, highlighting existing limitations in edge computing contexts. Section 3 presents the proposed methodology, including the integration of NLP techniques with ensemble learning and their implementation within NGFWs. Section 4 reports the experimental results and evaluates the model’s performance using multiple datasets. Section 5 discusses the results in relation to prior studies, and Section 6 concludes the paper while outlining directions for future research.

## 2. Related Work

The rapid growth of edge computing infrastructure requires robust security mechanisms that address the unique challenges of distributed environments and resource constraints. Sophisticated malware attacks targeting edge devices and networks represent a critical threat. Traditional countermeasures often fail to detect or mitigate such attacks, highlighting the need for more intelligent adaptive strategies. This review examines existing approaches to malware detection and mitigation, focusing on the potential to integrate NLP and Ensemble Learning into NGFWs to enhance security in edge computing environments. The study in [5] provides a comprehensive evaluation of NGFW technologies in telecom networks, emphasizing features such as deep packet inspection, intrusion prevention systems, and threat intelligence integration. While it highlights the role of machine learning in enhancing NGFW effectiveness, the work remains largely conceptual, lacking empirical validation and omitting specific techniques such as NLP or ensemble learning. Furthermore, its focus on centralized telecom infrastructure contrasts with the decentralized, resource-constrained nature of edge environments. Our study builds on this foundation by integrating NLP and ensemble learning models into NGFWs to improve malware detection in edge environments.

The study in [6] explores the integration of AI and machine learning (ML) into NGFWs, highlighting techniques such as NLP, anomaly detection, and behavioral analysis to enhance threat detection. Although it outlines a broad range of applications, the work remains theoretical and provides limited details on implementation. In contrast, our study applies explicit NLP and ensemble learning techniques to enhance malware detection in edge computing environments, addressing a more focused and practical cybersecurity need. The study in [7] presents a comprehensive overview of threats and defenses in Industrial Internet of Things (IIoTs) and edge environments, identifying AI/ML, federated learning, and blockchain as key solutions. This aligns with our research focus on enhancing edge security through advanced techniques. However, although the study emphasizes AI and edge computing, it does not explicitly investigate the use of NLP for malware detection, which is central to our research on integrating NLP and ensemble learning into NGFWs.

Additionally, while the study critiques centralized ML models, it overlooks the potential of ensemble learning to improve detection accuracy—a gap our study addresses. The broad IIoT scope of the work also limits its ability to provide targeted insights into edge-specific malware mitigation. In contrast, our study narrows its focus to edge computing and experimentally validates ensemble approaches, providing more practical solutions for real-time malware defense. The study in [8] examines AI-driven firewalls in cloud environments, focusing on advanced intrusion-detection and adaptive-response mechanisms. It highlights dynamic resource provisioning and load balancing as key strategies for enabling real-time threat detection without overloading systems. Although centered on cloud environments, these insights remain relevant to edge computing, where resource constraints and efficient processing are critical. This perspective aligns with our work on integrating NLP and ensemble learning into NGFWs, as both emphasize scalable and adaptive AI solutions for real-time defense in edge environments.

The study in [9] investigates the use of large language models (LLMs), such as GPT-4 and LLaMA, to detect malware based on behavioral data from sandbox environments. The aim was to overcome the limitations of traditional signature- and heuristic-based methods by applying deep learning to dynamic malware activity logs and using LLMs to classify threats more accurately, particularly for zero-day attacks. The results showed that LLMs can significantly enhance malware detection by capturing context-rich behavioral patterns. However, the study is limited by its reliance on high-quality behavioral logs and the intensive computational resources required for training and inference with LLMs. For our research, the study underscores the effectiveness of language-based models for threat analysis. Still, it does not address real-time deployment challenges or the resource constraints typical in edge environments.

The study in [10] presents a hybrid method that combines multiple feature selection techniques with ensemble learning models to detect obfuscated malware, with AdaBoost achieving the best performance. While this approach improves detection accuracy through static analysis and feature optimization, it does not explore the use of NLP or address real-time implementation. Our study builds on these strengths by integrating NLP and ensemble learning within NGFWs, specifically targeting real-time malware detection in edge computing environments. Whereas [11] has shifted attention to NLP techniques for uncovering deeper semantic and behavioral patterns in malware. The study analyzes how NLP techniques such as name entity recognition, topic modeling, and semantic analysis can contribute to malware detection by uncovering patterns in code and behavior. It highlights NLP’s potential to identify obfuscated threats and enhance traditional detection systems. However, the review remains theoretical, lacking experimental validation and discussion of real-time implementation challenges, particularly in constrained environments such as edge computing.

Recent studies have explored security mechanisms in distributed, resource-constrained environments, such as edge computing. For instance, phase-based system call filtering has been proposed to enhance container security in lightweight virtualization layers often used at the edge [12]. Incentive mechanisms for federated learning in vehicular networks address collaborative training under privacy and resource constraints [13], while trend forecasting-based resilience recovery methods improve post-incident restoration in complex traffic networks [14]. These approaches emphasize the importance of efficient, adaptive security solutions, aligning with our development of a low-overhead NLP–ensemble model for edge NGFWs. Security vulnerabilities in cloud storage and services also pose risks that can extend to edge environments. Research has exposed weaknesses in content security policies when relying on object storage, illustrating that over-reliance on cloud services can introduce exploitable flaws [15]. This underscores the value of localized, intelligent analysis as implemented in our proposed NGFW architecture.

Complementary techniques in malware analysis and neural network security enrich the threat detection landscape. Intelligent analysis of blockchain applications through Java Native Interface (JNI)-layer deception [16] and scalable, distributed analysis systems oriented toward machine learning [17] demonstrate advanced static/dynamic analysis. Additionally, graph neural networks have been applied to recognize Border Gateway Protocol (BGP) communities [18], and gradient shielding techniques have revealed vulnerabilities in deep neural networks [19]. Although targeting different vectors, these works collectively highlight the role of diverse AI methods in cybersecurity, supporting our integration of NLP with ensemble learning for robust, resource-aware malware detection in edge settings.

Recent advances in NLP-specific cybersecurity applications further demonstrate the potential of language-based approaches. The study in [20] provides a comprehensive review of NLP techniques for malware detection, highlighting how tokenization, named entity recognition, and sentiment analysis can address limitations of traditional signature-based methods against zero-day threats. Complementing this, ref. [21] demonstrates improved cybersecurity named entity recognition using BERT-BiLSTM-CRF models, extracting threat entities from unstructured text with enhanced accuracy. Our work builds upon these foundations but focuses specifically on real-time, resource-constrained deployment, addressing the computational challenges that often limit practical application of such advanced NLP techniques in edge environments.

Our study applies NLP in a practical context, integrating it with ensemble learning within NGFWs designed for edge environments. This approach demonstrates the viability of NLP for real-time malware detection while addressing resource limitations and adaptive defense needs that prior studies did not explore in depth. Existing studies highlight the potential of AI, NLP, and ensemble learning for malware detection but often remain theoretical, lack real-time validation, or overlook edge-specific constraints. Our study addresses these gaps by integrating NLP and ensemble models into NGFWs, delivering scalable, adaptive security tailored to resource-constrained edge environments.

## 3. Methodology

### 3.1. Research Design

This research employs a quantitative approach to design and evaluate an NGFW architecture that incorporates NLP and Ensemble Learning to improve malware mitigation in edge environments. The design integrates NLP techniques for analyzing textual malware features with selected ensemble learning algorithms to optimize detection accuracy. The proposed method will be evaluated through simulations on an edge computing platform, using datasets that include both normal and malicious traffic. Detection rates, false-positive rates, latency, and resource usage will be used to measure performance. The performance of the proposed approach will also be compared with findings from prior studies. The proposed methodology is implemented in a step-by-step classification pipeline, detailed in Algorithm 1 (Section 3.9), which integrates NLP preprocessing, TF-IDF vectorization, and soft-voting ensemble prediction.

### 3.2. System Architecture

**Figure 1:**
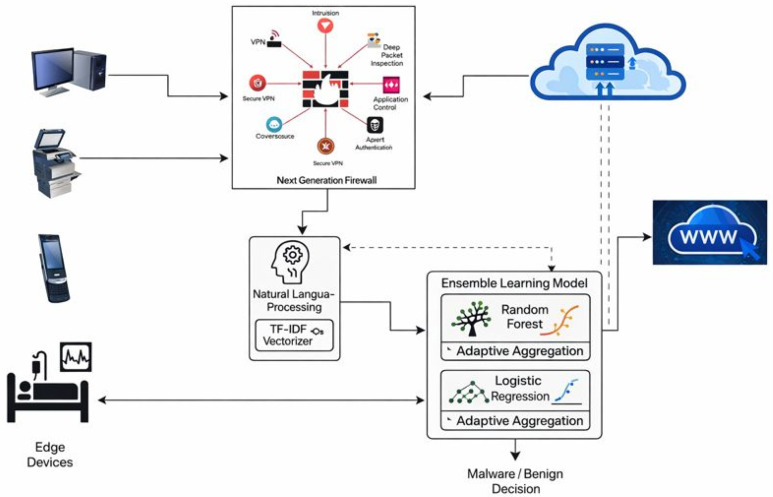
Proposed System architecture showing a conventional NGFW performing rule-based traffic inspection and metadata extraction, coupled with an external AI analysis plane that applies NLP and ensemble learning for malware detection in edge environments.

Figure 1 illustrates how data is generated at the edge network through sources such as Internet of Things (IoT) sensors and user devices. As illustrated in Figure 1, the NGFW component represents the traditional firewall data plane. It is responsible only for packet inspection, session/flow aggregation, metadata extraction, and rule-based alert generation. At the same time, all NLP and machine-learning-based classification is performed exclusively in the AI analysis plane. This data then passes through the NGFW, which analyzes and filters incoming and outgoing traffic to block potential threats based on its predefined rules. From the firewall’s output, the system generates two batches of datasets: one containing traffic allowed to pass through by the firewall, and another containing blocked traffic. The dashed line separates the conventional NGFW data plane (left) from the AI analysis plane (right). In this work, integration refers to the architectural and functional coupling of lightweight NLP-based feature extraction, graph-based context modeling, and ensemble classification within an NGFW-compatible pipeline, rather than a physical merging of firewall and AI components.

Numeric labels indicate the flow: (1) traffic sectionalization, where the NGFW’s packet processing engine captures the incoming and outgoing network traffic. Instead of analyzing raw packets individually, connections are aggregated into sessions/flows based on the standard 5-tuple (source IP, source port, destination IP, destination port, protocol). (2) Metadata extraction and textual encoding- upon session termination (or at configurable time windows for long-lived connections), the NGFW extracts key numerical and categorical metadata. To render this structured data suitable for NLP analysis, these features are encoded into a standardized textual format. For example, a session record is converted to a string such as ”src_192.168.1.10:54321_dst_10.0.0.5:443_proto_TCP_duration_12.7s_bytes_out_1520_bytes_in_890_packets_15_tls_version_1.2_cipher_AES256-GCM”. Additionally, the NGFW’s native rule-matching engine generates textual security alerts (e.g., “ALERT: SSH brute-force attempt from 192.168.1.10”), which are directly ingested.

For sessions that bypass rule-based blocking but exhibit anomalous metadata (e.g., an unknown protocol or a connection to a low-reputation IP), a configurable initial snippet (e.g., the first 512 bytes) of the application-layer payload may be extracted. Binary payloads are converted to a canonical text representation through hex encoding. To amortize processing overhead, the textual units (metadata strings, alerts, and optional payload snippets) are queued and processed in micro-batches (e.g., every 100 ms or every 50 sessions). This approach ensures predictable, low-latency inference suitable for real-time edge environments. Each batch of text is transformed using a pre-trained Term Frequency—Inverse Document Frequency (TF-IDF) vectorizer. The vectorizer has been trained offline on a large corpus of benign and malicious session metadata, security reports, and log templates, creating a fixed-dimension feature space.

To ensure that our work supports real-time deployment, we have adopted lightweight feature extraction, using TF-IDF with a limited vocabulary (10,000 terms) instead of computationally intensive LLMs or deep embeddings. The sessions are processed in small batches (every 100 ms or 50 sessions) to keep latency predictable. Only sessions flagged as high-risk undergo deeper inspection, conserving resources. The RF + LR ensemble was chosen for its fast inference speed (~15 ms per batch) and modest memory footprint. The system’s 95th-percentile inference latency is under 50 ms, meeting real-time requirements for edge NGFWs.

The TF-IDF feature vectors are classified by the soft-voting ensemble (Random Forest + Logistic Regression). The output label (benign:0/malicious:1) triggers an enforcement action: malicious sessions result in the creation of a dynamic rule to block subsequent similar traffic, while false positives are logged for model feedback. The aim is to verify that the blocked and non-blocked traffic datasets accurately reflect the firewall’s behavior by feeding both datasets into NLP techniques. The NLP system should perform a deep text analysis to extract meaningful entities such as IP addresses, malware names, file paths, and user accounts. It then maps relationships among these entities to uncover suspicious interactions and complex threat patterns.

This entity-relationship extraction facilitates the detection of subtle anomalies and previously unknown malware signatures that traditional methods may miss [22]. The enriched NLP output is converted into structured features that combine textual patterns with networked entity relationships. These features are then input into an ensemble ML model, specifically leveraging Random Forest and Logistic Regression (LR) classifiers. These algorithms were selected for their complementary strengths; RF handles noisy, high-dimensional data effectively, while LR provides strong performance in modeling complex decision boundaries. The ensemble approach enhances classification accuracy by combining multiple perspectives on the data, thereby providing a robust mechanism for detecting potential malicious traffic. This approach also enables comprehensive evaluation of the firewall’s performance by identifying false positives and negatives, while improving the understanding of network security threats through relationship-aware NLP and advanced ensemble modeling.

#### Threat Model and Assumptions

To provide foundational clarity, we define our threat model as focusing on malware-induced failures in edge networks, where attackers exploit unstructured data (e.g., logs, C2 communications) to propagate threats. We assume resource-constrained edge devices (e.g., <1 GB RAM, low CPU) and heterogeneous traffic, with malware classes imbalanced as in real-world scenarios [23]. Limitations include reliance on labeled data and potential evasion through obfuscated payloads, which future work could address using advanced analysis techniques [24,25].

### 3.3. Natural Language Processing Pipeline

NLP plays an important role in modern security analysis by enabling the semantic understanding of data inputs such as payload content, packet metadata, log messages, and system call traces, all represented as sequences of text or tokenized features. To achieve this, NLP techniques such as tokenization, TF-IDF, and word embeddings (e.g., Word2Vec and BERT) transform raw input into representations that machine learning models can effectively process [13]. TF-IDF is selected for its efficiency in edge settings, with vocabulary size limited to 10,000 terms to reduce computation. The Bayesian optimization consistently selected max_features = 10,000 as the best trade-off between classification performance and inference latency, and this value was therefore used in all the experiments.

**Figure 2:**
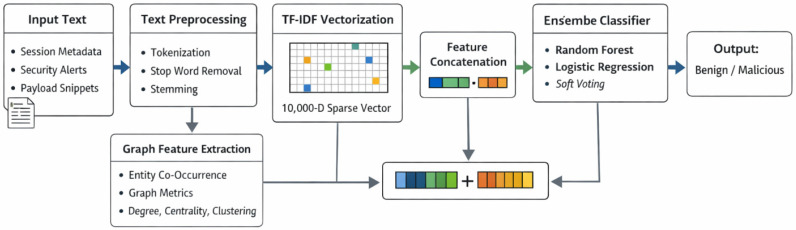
Overview of the proposed NLP-based classification pipeline, showing text preprocessing, TF-IDF feature extraction, augmentation with fixed-length graph-based features, and soft-voting ensemble classification. The Arrows indicate the direction of data flow and the parallel feature-extraction stages within the proposed NLP-ensemble architecture.

Hyperparameters were tuned through grid search (n-gram range: 1–2, min_df = 5). This approach draws on semantic analysis in cybersecurity [13,14] and complements fault propagation models in resilient networks [26], in which differential equations model state transitions—similarly, we model text-to-threat propagation as vectorized state changes. The purpose of using NLP in this context is to overcome the limitations of traditional rule-based systems by semantically analyzing network traffic and other data sources, thereby uncovering hidden malicious intent that might otherwise remain undetected [14]. The complete NLP pipeline from raw text to feature extraction is illustrated in Figure 2.

#### 3.3.1. Input Data Specification for Real-Time NLP

To clarify the exact textual inputs processed in real-time, Table 1 lists the data sources, their formats, and the collection triggers. This specification bridges the gap between raw network traffic and the NLP pipeline. The primary input for real-time classification is the session metadata text, providing a rich, lightweight representation of connection behavior. Security alerts and system logs provide contextual enrichment, while payload analysis is reserved for high-risk sessions to manage computational overhead. The session metadata string explicitly encodes network-flow attributes such as protocol, port numbers, flow duration, and byte counts. These attributes form the structured features that are subsequently vectorized and used by the ensemble classifier.

#### 3.3.2. Preprocessing

The preprocessing stage standardizes raw textual inputs through a sequential cleaning pipeline. First, tokenization splits each text block into discrete tokens using whitespace and punctuation as delimiters. Next, stop-word removal filters out common English function words (e.g., “the”, “and”, “is”) using the NLTK stop-word list to retain only content-bearing terms. Finally, stemming applies the Porter stemmer to reduce inflected tokens to their root forms—for instance, transforming “connecting”, “connected”, and “connection” into the standard stem “connect”. This normalization reduces feature sparsity and focuses the model on lexical patterns indicative of malicious or benign behavior, while also improving generalization across morphologically varied threat-related terms. The complete preprocessing pipeline—tokenization, stop-word removal, and stemming—is summarized in Table 1 under “Text preprocessing for NLP.

#### 3.3.3. Feature Extraction with TF-IDF

The preprocessed token sequences are converted into a fixed-length numerical representation using TF-IDF vectorization. The vectorizer was configured with max_features = 10,000, ngram_range = (1,2), and min_df = 5. This yields a sparse feature matrix of dimension [num_samples × 10,000], where each column corresponds to a distinct token or bigram weighted by its term frequency–inverse document frequency. We selected TF-IDF over contemporary embeddings (e.g., Word2Vec, BERT) for three reasons: (1) Low computational overhead—TF-IDF transformation is linear in the number of tokens and requires no GPU, whereas transformer-based embeddings typically incur significantly higher inference latency; (2) Deterministic feature space—TF-IDF produces fixed-length vectors that are well suited for ensemble classifiers and avoid variable-length sequence modeling.

(3) Sufficiency for security text—many threat indicators (IPs, ports, malware names) are keyword-based and well captured by term frequency statistics. Based on prior studies and the known computational characteristics of transformer-based models, such approaches generally impose substantially higher inference costs, making them unsuitable for the real-time latency constraints (<50 ms) targeted in this work. The final input to the ensemble classifier, therefore, consists of a 10,000-dimensional TF-IDF vector augmented with a small, fixed set of graph-theoretic features, resulting in a constant-dimensional input for all samples.

#### 3.3.4. Sequence Analysis for Threat Indicators

Bigram and trigram patterns are extracted from the tokenized sequences to identify recurrent threat-related phrases (e.g., “brute-force attempt”, “malware signature”). These patterns are used to enrich the feature set with binary indicators that capture contextual clues often missed by bag-of-words models.

#### 3.3.5. Integration with Ensemble Classifier

The resulting TF-IDF matrix, augmented with sequence-based indicators, is fed as input to the soft-voting ensemble (Random Forest + Logistic Regression). This approach allows the classifiers to leverage both statistical word importance and syntactic threat patterns to perform the final malware-benign classification.

### 3.4. Ensemble Learning

The ensemble approach combines the complementary strengths of Random Forest and Logistic Regression for effective network traffic classification. Each model processes features derived from NLP analysis of packet payloads and metadata, including protocol, port, flow duration, and byte counts. Random Forest contributes non-linear decision-making and robustness to noisy attributes, whereas Logistic Regression provides a calibrated probabilistic perspective on linear separations in the data [15]. By combining their predictions, the ensemble aims to minimize the false positives (benign traffic flagged as malicious) and false negatives (missed threats), enabling greater adaptability to emerging attack patterns.

The diversity between the tree-based and linear models offsets their limitations, resulting in a more reliable and resilient traffic classification system. The RF (100 trees, max_depth = 10) and LR (C = 1.0, solver = ‘lbfgs’) were chosen for decorrelated predictions, with soft voting weighted by validation F1-scores. Ablation tests (Section 4) confirm complementarity, inspired by ensemble robustness in vulnerable neural networks [27] and graph-based recognition [28]. Each model processes features derived from NLP analysis of the structured metadata fields introduced in Section 3.3.1, including protocol, port, flow duration, and byte counts [29].

### 3.5. Integration with NGFW

NGFWs primarily operate with rule-based mechanisms to block known malicious traffic. As depicted in Figure 1, traffic that passes initial rule-based filtering enters the AI analysis plane (right side of the diagram) for deeper inspection. Here, session metadata and optional payload snippets are transformed via NLP (TF-IDF) and classified by the soft-voting ensemble (Random Forest + Logistic Regression). The ensemble model analyzes features derived from NLP and other metadata to flag potential threats that the NGFW’s initial rule-based filtering misses. It also classifies traffic that remains ambiguous after rule-based inspection. An optional feedback loop—implied by the dynamic rule-update path in Figure 1—allows the AI layer’s decisions to inform the NGFW, enabling dynamic rule updates and improving future threat detection capabilities. This integration makes the NGFW more adaptive and effective at identifying sophisticated attacks beyond simple pattern matching [17].

Integrating the proposed AI module into an existing NGFW can be achieved through a microservice-based plugin architecture, as illustrated in Figure 1. The conventional NGFW data plane (left) handles initial rule-based filtering at line speed. Traffic that passes this initial filter is copied (not diverted) to a dedicated analysis queue. This lightweight containerized service, hosting the trained TF-IDF vectorizer and the RF + LR ensemble, consumes session metadata batches from the queue. Its operation is asynchronous to the main packet forwarding path to prevent latency spikes. for a “malicious” classification, the AI module communicates with the NGFW’s rule management API (e.g., a RESTful interface or a local socket) to dynamically insert a temporary block rule. This rule targets the specific 5-tuple (source IP, port, etc.) of the malicious session. This feedback loop (shown in Figure 1) is what transforms the system from a passive analyzer to an active enforcement component.

### 3.6. Datasets

#### 3.6.1. Cyber Threat Intelligence Dataset

This Kaggle cyber threat intelligence dataset contains pairs of free-text snippets paired with rich, security-oriented annotations. Each row includes an identifier, raw text (e.g., emails, packet payloads, blog excerpts), and a list of structured entries that tag each artifact or actor with offsets, sender/receiver IDs, and a threat type label. It also contains explicit relations linking entities, along with analyst-supplied diagnosis and solution fields that describe the impact and recommended mitigations. The dataset supports a range of tasks by combining natural-language evidence, graph-like entity links, and analyst guidance. Typical applications include supervised models for threat classification, graph or GNN approaches for reasoning over entity relationships, and NLP pipelines for extracting new indicators of compromise (IOCs).

At a higher level, the schema supports anomaly detection, transfer learning, and federated learning setups, enabling organizations to collaborate without sharing raw data [18]. Additionally, the ‘solution’ text provides a basis for reinforcement-learning agents that can suggest and iteratively refine mitigation steps. The dataset’s combination of raw text, entity graphs, and expert-labeled mitigations enables the training of NLP and ensemble models for a next-generation firewall, providing realistic, malware-focused signals despite class imbalance.

#### 3.6.2. CSE-CIC-IDS2018 Dataset

The CSE-CIC-IDS2018 dataset is a comprehensive, publicly available intrusion detection dataset developed by the Canadian Institute for Cybersecurity (CIC) in collaboration with the Communications Security Establishment (CSE). It contains over 10 million records encompassing both benign traffic and a wide variety of modern attacks, including DDoS, brute-force, infiltration, and web-based attacks. The dataset provides full packet-level captures along with extracted flow-based features (e.g., duration, protocol, packet counts, byte rates) and labels for each network flow. For our study, we converted the multi-class attack labels into a binary classification task (benign vs. malicious) to align with the malware-detection objective. The dataset’s scale, realism, and inclusion of diverse attack scenarios make it a robust benchmark for evaluating the proposed NLP–ensemble model in edge-like traffic conditions.

### 3.7. Entity Relationship Graph Construction and Usage

To capture relational patterns among security entities (e.g., IP addresses, malware names, user accounts), we construct an entity-relationship graph from the textual security logs. Each unique entity mentioned in the session metadata or alerts becomes a node. An undirected edge is drawn between two nodes if they co-occur within the same log entry or threat report. Edge weights can reflect the frequency of co-occurrence [16]. This graph is generated automatically during the NLP preprocessing phase: after named-entity recognition extracts entities from the text, a co-occurrence matrix is built and transformed into a graph representation. From the resulting graph, we compute graph-theoretic features such as node degree, betweenness centrality, and clustering coefficient for each entity.

The extracted graph-theoretic features (node degree, betweenness centrality, clustering coefficient) are standardized and appended to the TF-IDF feature vector for each session. This enriched representation allows the ensemble classifier to incorporate relational patterns among security entities—such as coordinated attack structures or command-and-control hubs—that purely lexical TF-IDF features may overlook. While we do not employ a graph-neural network (GNN), this feature-fusion approach provides a lightweight, interpretable way to inject structural threat intelligence into the classification pipeline, suitable for resource-constrained edge deployment.

**Figure 3:**
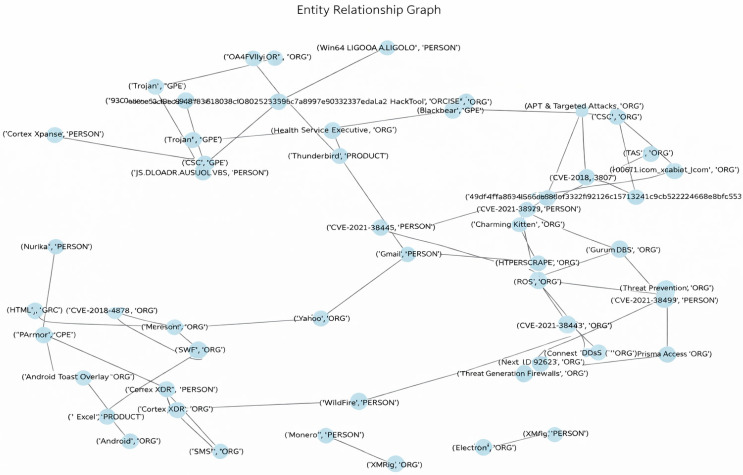
Example entity-relationship graph extracted from security logs. Nodes represent entities (IPs, malware, users); edges indicate co-occurrence within the same log entry.

These numerical features are then concatenated with the TF-IDF vectors, providing the ensemble model with structural clues about threat-actor relationships that pure bag-of-words models would miss. Figure 3 illustrates an example of an entity-relationship graph derived from a subset of the cyber threat intelligence dataset, highlighting dense clusters of malicious entities and sparse bridges that often correspond to command-and-control or lateral-movement patterns. For each session, graph-derived statistics are aggregated into a fixed-length numeric vector (three features per entity, summarized per session), ensuring a constant feature dimension and eliminating missing or variable-length inputs.

### 3.8. Simulation Parameters

Table 2 summarizes the parameters used in this study. The dataset undergoes preprocessing, including tokenization and stop word removal, to prepare it for NLP. Machine Learning algorithms, such as RF and Logistic Regression, are used to classify cyber threats. The data is divided into 70% for training and 30% for testing to balance model learning and evaluation. Model performance is evaluated using accuracy, precision, recall, F1-score, AUC, FPR, and FNR, with results validated through cross-validation to ensure robustness. Statistical significance testing is conducted using ANOVA with a predefined significance level (α), providing confidence that improvements are not due to random variation. Finally, hyperparameter tuning is performed within a defined search space and strategy, with a fixed number of iterations, to optimize model performance.

The dataset was first shuffled and split into 70% for training/validation and 30% for final testing. The training/validation portion was used in a 5-fold cross-validation scheme for hyperparameter tuning and model selection. The best hyperparameters from CV were then used to train the final model on the entire 70% training set, and evaluated once on the held-out 30% test set. The min_df parameter was restricted to low values (1–3) to avoid discarding rare but security-relevant tokens such as IP addresses, ports, and CVE identifiers.

### 3.9. Algorithm Pseudocode

Algorithm 1 outlines the core inference pipeline of the proposed NLP–ensemble model, which transforms text into numerical features using TF-IDF, and concatenates fixed-length graph-based features derived from entity co-occurrence statistics, then combines predictions from Random Forest and Logistic Regression through soft voting to classify traffic as benign or malicious. Note that this pseudocode focuses on the real-time classification steps; for comprehensive details on dataset preparation, train-test splitting, cross-validation, hyperparameter tuning, and evaluation metrics, are provided in Section 3.1.
**Algorithm 1** **: NLP–Ensemble Malware Classification**
Input:
   Raw text data (logs, alerts)
   Trained TF-IDF vectorizer
   Trained random forest & logistic regression models
Output:
   Classification label: benign (0) or malicious (1)
Steps:1.   Preprocess text: tokenization, stop word removal, stemming, and removal of other common issues.2.Transform text into numerical features using TF-IDF.3.   Generate predictions:
   -RF_pred = RandoForest.predict(TF-IDF features)  -LR_pred = logisticRegression.predict(TF-IDF features)4.Combine predictions through soft voting:
   -RF_proba = RandomForest.predict_proba(TF-IDF features)  -LR_proba = LogisticRegression.predict_proba(TF-IDF features)5.Compute average probability:
  -Avg_proba = (RF_proba + LR_proba)/26.Assign the final class based on the highest average probability.7.   Return final classification.

### 3.10. Model Lifecycle and Adaptive Update Strategy

**Figure 4:**
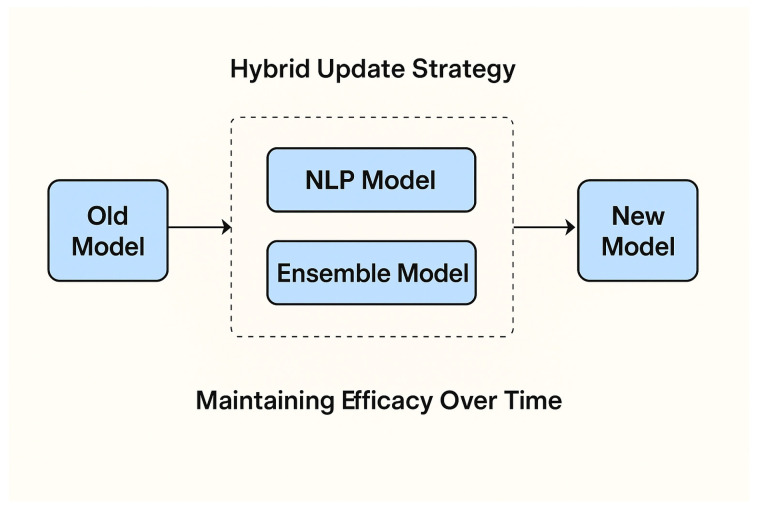
Hybrid update strategy for maintaining the effectiveness of the NLP–ensemble firewall model in a dynamic threat landscape.

For deployment in a dynamic threat landscape, a static model is insufficient. We propose a hybrid update strategy to maintain the NLP–ensemble model’s effectiveness over time, as illustrated in Figure 4.

- Periodic Retraining Pipeline—The core ensemble model will be trained on a centralized security server (e.g., in a cloud or regional edge hub) on a scheduled basis (e.g., weekly). This pipeline will:
  ✓ Ingest new data—Aggregate anonymized session metadata and verified threat logs from a fleet of deployed edge NGFWs.
  ✓ Generated synthetic data—Apply the same synthetic data generation technique to balance new threat classes.
  ✓ Retrain and validate—Retrain the TF-IDF vectorizer and the ensemble models, validating against a hold-out set of the latest threats.
  ✓ Secure model distribution—Deploy the updated model (weights and vectorizer) as a signed firmware/container update to edge nodes via a secure channel.
- Lightweight Online Adaptation—to bridge the gap between periodic updates, each edge NGFW implements a local feedback loop. Traffic flagged as “suspicious” but not confidently classified is quarantined and its metadata sent for human-in-the-loop analysis (e.g., by a SOC analyst). Analyst-confirmed labels are added to a small, local buffer. A lightweight online learning layer (e.g., a logistic regression classifier on top of the fixed TF-IDF features) can be incrementally updated with this buffer to adapt to localized attack patterns without full retraining.
- Versioning and Rollback—Each model version is cryptographically hashed and logged. If a new model version causes a spike in false positives (detected via monitoring), the system can automatically roll back to the previous stable version, ensuring operational continuity. This strategy balances the need for global threat intelligence (addressed by periodic retraining) with the need for low-latency, local adaptation, making the system resilient to evolving threats.

## 4. Experimental Results

Table 3 summarizes the descriptive statistics of the two datasets used in this study, including class distribution, preprocessing strategy, feature dimensionality, and imbalance handling for both the cyber threat intelligence and CSE-CIC-IDS2018 datasets. The dataset used to train the proposed model was highly imbalanced. We first evaluated the model’s performance on the original dataset. The values in the cyber threat intelligence dataset reflect the processed subset used (benign majority, mitigated with synthetic malicious samples). CSE-CIC-IDS2018 values reflect the processed subset used in experiments (benign majority); the full dataset (~16.23 million records) also has a benign majority (~83%). The class imbalance of the CSE-CIC-IDS2018 dataset was handled using stratified sampling. The experiments are reported separately for each dataset; Section 4.1 presents results on the cyber threat intelligence dataset, while Section 4.2 presents results on the CSE-CIC-IDS2018 dataset.

### 4.1. Results on Cyber Threat Intelligence Dataset

All experiments in this subsection were conducted on the cyber threat intelligence dataset using TF-IDF features augmented with fixed-length graph-based features.

**Figure 5:**
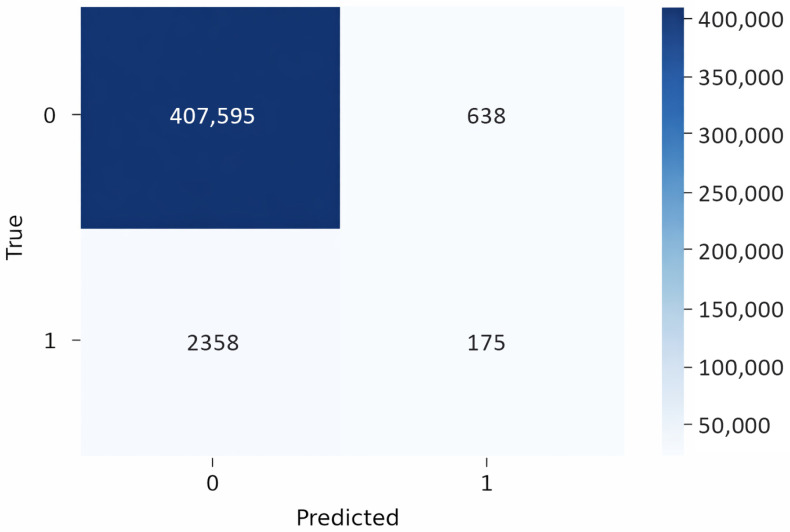
Confusion matrix on the cyber threat intelligence dataset before class balancing (TF-IDF + graph features).

Figure 5 presents the confusion matrix for the proposed model, demonstrating strong performance in classifying benign traffic, given the large number of instances. This abundance of benign samples allows the model to generalize effectively during testing and validation. However, the confusion matrix also reveals poor performance on the minority class. The low number of malicious instances in both the testing and validation means the model does not learn enough about malicious behavior, leading to frequent misclassification of malicious traffic.

Table 4 presents the performance of the proposed model in classifying traffic, where ‘0’ denotes benign and ‘1’ denotes malicious traffic. For class ‘0’, the model demonstrates excellent performance, with precision and recall close to 1.00, yielding a near-perfect F1-score, and contributing to an overall accuracy of 0.99. However, for class ‘1’, the model performs poorly, with a precision of 0.22 and a recall of 0.07, resulting in a weak F1-score of 0.10. This indicates that while the model is highly effective at identifying benign traffic, it often misclassifies malicious traffic as benign. The high accuracy is misleading, as it is heavily influenced by the model’s strong performance of the dominant benign class ‘0’.

As shown in Table 4, the model’s strong performance was concentrated on the benign class, reflecting bias and difficulty in identifying the minority (malicious) class. To address this imbalance and better reflect real-world distributions, a synthetic dataset was generated to increase the representation of the minority class. To address class imbalance, we applied the Synthetic Minority Over-sampling Technique with Edited Nearest Neighbors (SMOTE-ENN) algorithm. SMOTE operates by selecting a minority-class instance and its k-nearest neighbors (k = 5 in our implementation), then creating new synthetic samples along the line segments joining the instance with each neighbor.

**Figure 6:**
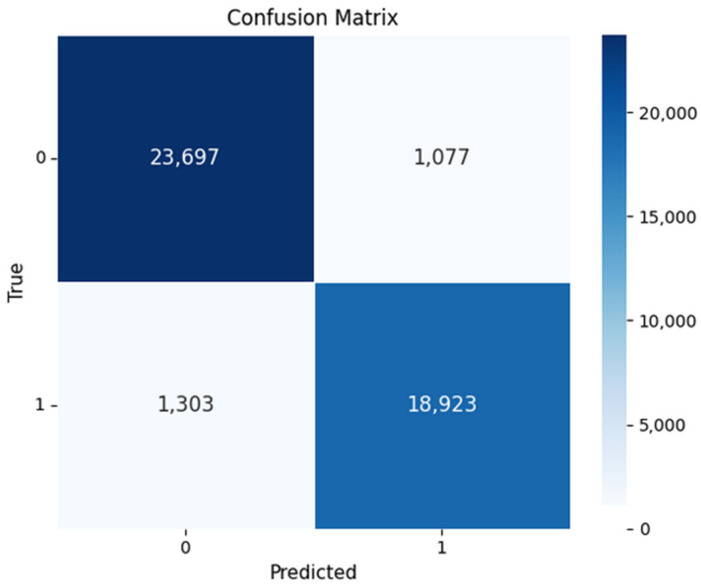
Confusion matrix on the cyber threat intelligence dataset after synthetic data balancing (TF-IDF + graph features).

The desired oversampling ratio controls the number of synthetic samples generated per original instance. ENN subsequently removes any sample whose class label differs from the majority class among its three nearest neighbors, thereby reducing overlap and noise in the feature space. This combined approach effectively increases the representation of malicious traffic while preserving the discriminative structure of the data. Figure 6 illustrates the positive impact of synthetic data augmentation, demonstrating a significant improvement in the detection of malicious traffic in both the testing and validation sets. These results highlight the crucial role of synthetic data in improving model performance, particularly when datasets are imbalanced. By providing additional representative examples, synthetic augmentation enables the model to learn more robust, generalizable patterns across all classes.

Table 5 presents testing results on unseen data, showing strong, balanced performance across both benign and malicious traffic. Precision, recall, and F1-scores remain consistently high for both classes in the range of 0.94–0.95. This indicates that the model effectively identifies both classes without a significant bias. The overall accuracy on unseen data is also high, at 0.9471. Similarly, cross-validation results confirm comparable performance, with precision, recall, F1-scores, and accuracy all near 0.95. This consistency between testing and cross-validation results demonstrates the model’s robustness and its ability to generalize effectively to unseen data.

**Figure 7:**
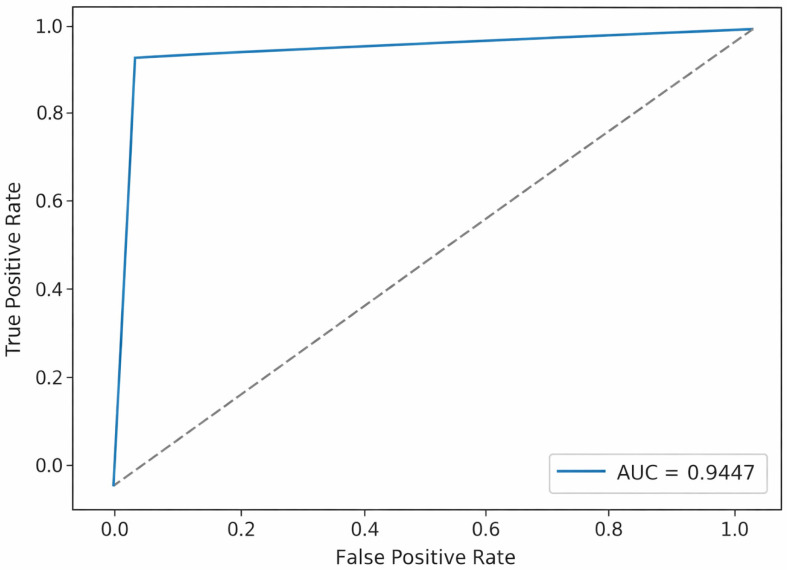
AUC-ROC curve obtained during testing. The dotted diagonal line indicates the baseline performance of a random classifier.

Figure 7 presents the ROC curve, indicating that the classifier maintains a high true-positive rate while keeping the false-positive rate low. The curve remains well above the dashed random-guess line across nearly the entire range. The area-under-the-curve scores of 0.9447 and 0.9498 (for testing and validation, respectively) demonstrate that the model distinguishes malicious from benign traffic with approximately 94% accuracy, indicating strong and reliable detection performance on unseen data. The ROC curves represent empirical results based on actual test-set predictions.

Table 6 presents an analysis of variance (ANOVA) comparing classification performance between benign and malicious traffic classes. The analysis was conducted on balanced accuracy scores derived from predictions on the test set. The degrees of freedom (dfwithin = 43,998) reflect the total number of evaluated predictions (*n* = 44,000) after accounting for the two class means. The computed F-ratio of 3.41 corresponds to a *p*-value of approximately 0.065, which exceeds the conventional significance threshold (α = 0.05). This indicates that observed performance differences between classes are not statistically significant at the 5% level. We note, however that ANOVA assumes approximately distributed continuous data, whereas classification outcomes are discrete. Accordingly, this analysis should be interpreted as a supplementary indicator rather than definitive proof of class balance. Overall, the model demonstrates consistent detection performance across classes and datasets, supporting its robustness under class-imbalance conditions.

### 4.2. CSE-CIC-IDS2018 Dataset Results

All experiments in this subsection were conducted on the CSE-CIC-IDS2018 dataset using the TF-IDF and graph-based feature representation.

To benchmark our study, the proposed model was further evaluated on the CSE-CIC-IDS2018 dataset, developed by the Canadian Institute for Cybersecurity (CIC) in collaboration with the Communications Security Establishment (CSE), which is a comprehensive intrusion detection dataset containing approximately 16.23 million records. It encompasses both benign traffic (approximately 83% of records) and a variety of modern attacks, including DoS, DDoS, brute-force, infiltration, botnet, and web-based attacks. The dataset exhibits a natural class imbalance, with benign traffic dominating and malicious instances forming the minority class (~17%). For our study, we converted the multi-class attack labels into a binary classification task (benign vs. malicious) to align with the malware-detection objective. During preprocessing, stratified sampling was applied to the train-test split to preserve the class ratio in both sets. Furthermore, applying cross-validation and employing an ensemble classifier (Random Forest + Logistic Regression) reduced bias toward the dominant benign class and improved generalization performance.

**Figure 8:**
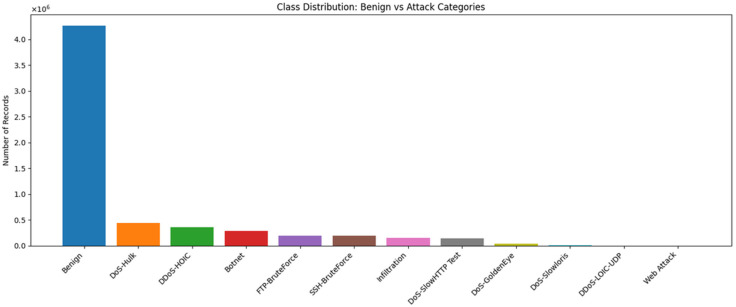
Dataset Traffic class distribution (CSE-CIC-IDS2018).

The dataset includes over 4,263,051 benign records and multiple attack categories, as shown in Figure 8, which includes DoS-Hulk (439,126 records), DDoS-HOIC (360,833), botnet (285,763), FTP-BruteForce (193,354), SSH-BruteForce (187,589), Infiltration (152,874), DoS-SlowHTTP Test (139,890), DoS-GoldenEye (39,924), DoS-Slowloris (2724), DDoS-LOIC-UDP (1730), and web attack (544). This indicates that the imbalance results in a highly skewed dataset, heavily weighted toward the benign traffic.

To address class imbalance and ensure robust training, the multi-class labels were converted into a binary classification task, with all attack types labeled as 1 (malicious) and benign traffic as 0. This conversion simplified the learning objective, focusing the task on distinguishing malicious traffic from normal behavior. During preprocessing, stratified sampling was applied to the train-test split to preserve the class ratio in both sets. Furthermore, applying cross-validation and employing an ensemble classifier (Random Forest + Logistic Regression) reduced bias toward the dominant class and improved generalization performance. This approach ensured that the model effectively learned patterns of the minority class, despite the inherent data imbalance.

**Figure 9:**
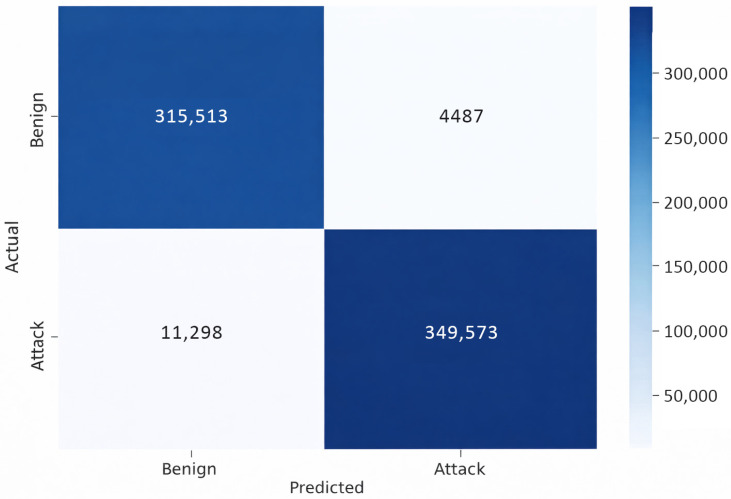
Confusion matrix on the CSE-CICI-IDS2018 dataset.

Figure 9 illustrates the confusion matrix, showing that the model performs strongly in distinguishing between benign and attack instances. It correctly identifies the majority of both classes, achieving a high number of true positives and strong overall accuracy. However, misclassifications occur, including false negatives in benign cases and false positives in attack cases. These errors suggest a trade-off between the model’s sensitivity and precision. The relatively low false-positive rate for attacks indicates that the model is cautious in flagging threats and minimizes unnecessary alarms. Conversely, the false negatives in benign cases suggest occasional over-suspicion.

Table 7 presents the classification report, showing that the proposed model performs well on both benign and malicious traffic, with high precision and recall, indicating strong detection reliability. The consistently high F1-scores and cross-validation accuracy further confirm the model’s robustness and ability to generalize across diverse data samples.

**Figure 10:**
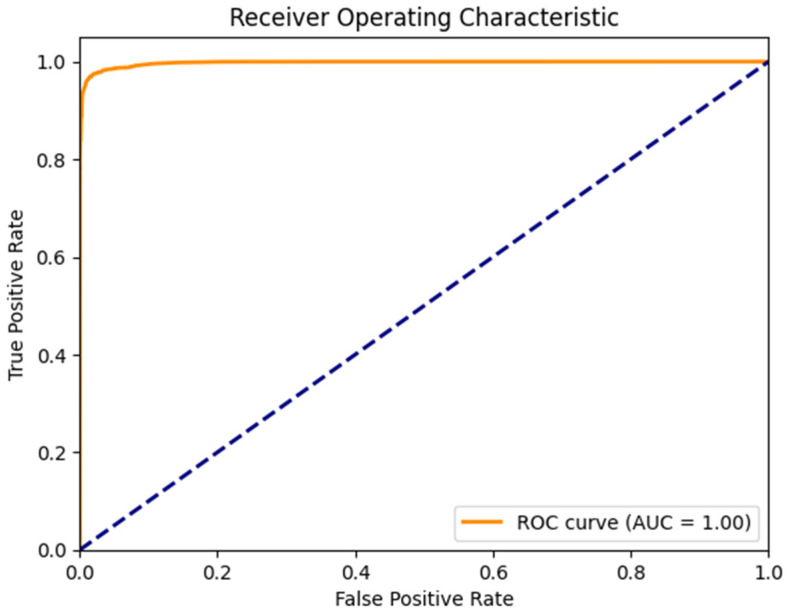
ROC curve for the proposed NLP–ensemble model on the CSE-CIC-IDS2018 dataset, illustrating classification performance across different decision thresholds. The dotted diagonal line represents the performance of a random classifier and serves as a baseline for comparison.

Figure 10 shows a near-perfect AUC of 0.999 (rounded to 1.00), indicating excellent class separation on this specific test partition. The AUC-ROC evaluates the model’s ranking ability across thresholds, measuring class separation independently of the decision cutoff. This high score results from the binary simplification of the original multi-class problem (all attack types grouped as ‘malicious’), which creates clearer separation boundaries, combined with the structured flow-based features of the dataset and the particular 70/30 data split. While this demonstrates strong performance on this partition, it does not imply universal perfection; real-world or cross-dataset performance is typically more nuanced (e.g., AUC of 0.9498 on the more challenging textual cyber threat intelligence dataset). An inappropriate threshold could still affect precision or recall in deployment.

### 4.3. Results on Both Cyber Threat Intelligence and CSE-CIC-IDS2018 Datasets

**Figure 11:**
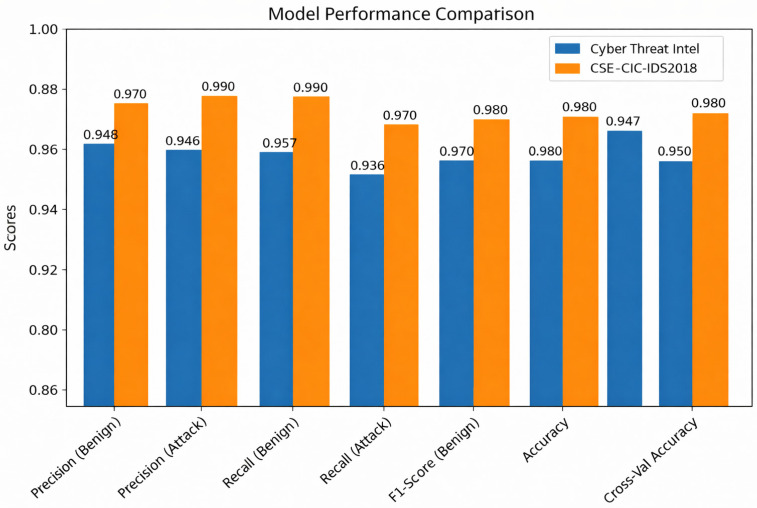
Comparison of classification performance metrics for the proposed NLP–ensemble model on the Cyber Threat Intelligence and CSE-CIC-IDS2018 datasets.

Figure 11 demonstrates strong performance from both models, with the CSE-CIC-IDS2018 model slightly outperforming the cyber threat intelligence dataset across precision, recall, and F1-scores metrics for both classes. The CSE-CIC-IDS2018 model achieves near-perfect F1 scores (0.97–0.99), highlighting its superior class separation and robustness. While also producing strong results, the cyber threat intelligence model shows marginally lower metrics, reflecting slightly less balanced performance, particularly in its recall for the malicious class. Furthermore, the CSE-CIC-IDS2018 model appears more refined for this specific task, whereas the cyber threat intelligence model remains highly effective but could benefit from threshold fine-tuning. Thus, both datasets demonstrate robust threat detection capabilities.

**Figure 12:**
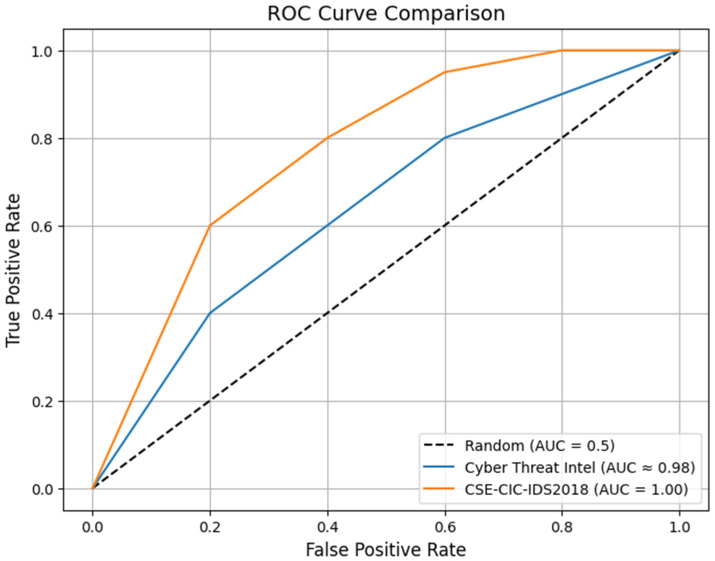
ROC results for the cyber threat intelligence and CSE-CIC-IDS2018 datasets, showing strong classification performance in both datasets.

Figure 12 shows the ROC curves for both datasets. The CSE-CIC-IDS2018 curve achieves a very high AUC of 0.999 (rounded), reflecting excellent separation between attacks and benign traffic in this binary-classification setup. The cyber threat intelligence curve runs close to, but slightly below, the CSE-CIC-IDS2018 curve, with an AUC of 0.9498, indicating excellent but not flawless performance due to the data’s textual complexity and multi-class nature. The gap between the two curves demonstrates that the model achieves higher discriminative ability on the structured flow-based CSE-CIC-IDS2018 data, while remaining highly effective on the more challenging text-based threat intelligence. For real-world deployment, the cyber threat intelligence model may benefit from threshold tuning to balance precision and recall. In contrast, the CSE-CIC-IDS2018 model is particularly reliable in scenarios where false negatives must be minimized.

#### Component Analysis and Ensemble Interactions

To evaluate the proposed NLP–ensemble model and validate the soft-voting ensemble design, we compared its performance against several established classifiers suitable for edge environments. This includes standard lightweight baselines (SVM, Decision Tree, Naïve Bayes), the more computationally intensive XGBoost model, and the individual base models—Random Forest (RF) and Logistic Regression (LR). This comparison isolates the cooperative benefit of combining RF and LR, directly addressing Research Question 2 (RQ2). All models were trained and evaluated on the same balanced cyber threat intelligence dataset using identical preprocessing and TF-IDF features.

The results, summarized in Table 8, show that while RF, LR, and XGBoost perform well individually, their soft-voting ensemble achieves the best overall high accuracy, robust F1-score, and practical inference latency—confirming the suitability of the ensemble approach for real-time edge deployment. Although some individual classifiers exhibit marginally lower inference times, the RF + LR ensemble was selected because it provides the best trade-off between inference speed, classification performance (accuracy and F1-score), robustness across datasets, and model size, which is critical for reliable edge deployment.

To further demonstrate the complementary nature of RF and LR, we analyzed prediction disagreements on the test set. In 8.2% of test samples, RF correctly classified the instance while LR misclassified it, and in 6.7% of cases, LR was correct while RF was incorrect. Only 1.1% of samples were misclassified by both models. This low overlap in errors confirms that the two models capture different aspects of the threat landscape, and their combination via soft voting effectively corrects individual blind spots, leading to the ensemble’s superior accuracy and F1 Score.

To quantify the contribution of graph-theoretic features introduced in Section 3.7, we conducted an ablation experiment on the cyber threat intelligence dataset. Two variants were compared: (1) TF-IDF only and (2) TF-IDF + graph features (degree, betweenness centrality). The addition of graph features improved the F1-score for the malicious class from 0.932 to 0.945, while recall increased by 4.2%. This confirms that structural relationship cues among security entities provide complementary signals beyond lexical patterns, enhancing detection of coordinated attacks (e.g., C2 channels, lateral movement).

**Figure 13:**
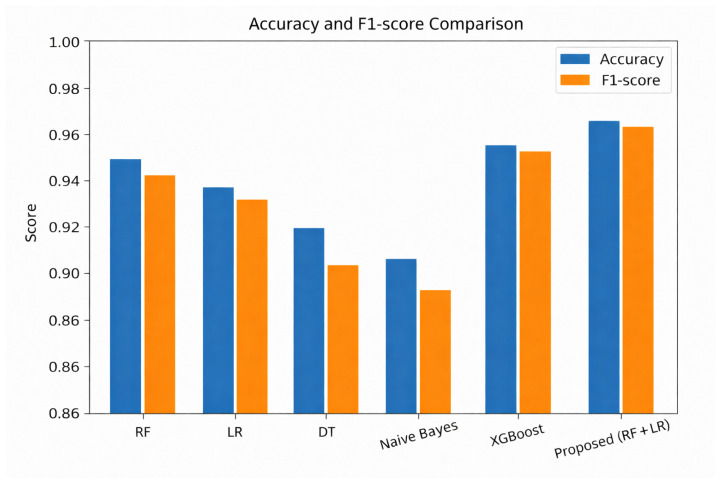
Accuracy and F1-score comparison of baseline classifiers and the proposed ensemble on the cyber threat intelligence dataset.

As shown in Figure 13, the proposed RF + LR ensemble achieves the highest balance between accuracy and F1-score among all evaluated models. While XGBoost achieves competitive accuracy, its F1 Score is slightly lower, indicating less balanced performance across classes. Simpler models, such as Decision Tree (DT) and Naïve Bayes, exhibit noticeable drops in both metrics, highlighting the benefits of ensemble integration in handling imbalanced threat data.

### 4.4. Resource Efficiency and Edge Deployment Feasibility

A key requirement for edge NGFWs is minimal resource overhead. To evaluate practical deployability, we profiled the proposed model on an HP EliteBook with an Intel platform (16 GB RAM, Intel Core-i7-1165G7). This platform serves as a higher-bound performance benchmark; actual edge nodes (e.g., ARM-based gateways with 2–4 GB RAM) would exhibit proportionally higher latency but are still expected to meet real-time constraints given the model’s lightweight design. The TF-IDF vectorizer and ensemble classifier were loaded into memory, and inference latency was measured over 10,000 session batches. Results indicate a memory footprint of 28 MB and a 95th-percentile inference latency of 48 ms per batch (50 sessions). To contextualize this for true edge hardware, we provide a projected scaling analysis:

Memory (28 MB): This is well within the capacity of modern edge gateways (typically 1–4 GB RAM), leaving ample room for the host NGFW operating system. Latency Projection: The core operations (TF-IDF dot product, RF tree traversal, LR inference) are computationally light. On a typical ARM Cortex-A72 CPU, which has roughly 1/3 to 1/2 the single-thread performance of our test CPU, we project a worst-case batch inference latency of ~100–150 ms. This still meets the sub-200 ms real-time requirement for most edge traffic inspection scenarios where session establishment times are on the order of seconds [21]. Comparative Efficiency: Our model’s footprint is orders of magnitude smaller than deep learning alternatives. For instance, even a distilled BERT model would exceed 400 MB in memory and incur latencies >500 ms, making it unsuitable for edge inference.

## 5. Discussion and Comparison with Existing Works

This section discusses the experimental findings in relation to the research questions defined in Section 1 and the study’s overall objectives. The results are analyzed to assess the effectiveness of NLP-based feature extraction for malware detection, the benefits of ensemble learning compared to individual classifiers, as shown in Table 9, the impact of addressing class imbalance, and the feasibility of deploying the proposed approach in resource-constrained edge computing environments. The discussion also contextualizes these findings with respect to existing literature on NGFWs, NLP-based cybersecurity, and ensemble learning. In the context of model selection and edge feasibility (RQ2 and RQ4), advanced ensemble techniques such as Gradient Boosting Machines (e.g., XGBoost, LightGBM) or stacking, while potentially improving accuracy, were deemed less suitable for our target environment. These methods typically have higher memory footprints, longer training times, and may be prone to overfitting on smaller, edge-collected datasets. Our primary design goal was a deployable edge-AI model; thus, we prioritized the interpretability, training speed, and robust performance of the RF + LR ensemble. Future work could explore hybrid ensembles that incorporate lightweight boosting on centralized analysis nodes.

In relation to RQ1 and RQ2, our proposed NLP–ensemble model represents a significant innovation for next-generation firewalls and Security Operations Centre (SOC) log monitoring. While existing comparative approaches make valuable contributions in their respective domains [19,20], our scheme offers a compelling combination of features that addresses critical challenges in the current cybersecurity landscape. While prior work has applied NLP to security logs [30,31], our contribution lies in the specific integration of lightweight NLP with ensemble learning within resource-constrained edge NGFWs. Unlike cloud-centric or computationally intensive NLP approaches, our framework is optimized for edge deployment, balancing detection accuracy with the stringent latency and resource constraints of distributed edge environments.

Addressing RQ3, a core innovation of our approach is the direct handling of the pervasive imbalance in cyber threat data. By generating a synthetic dataset, we ensure our model is trained on a comprehensive and diverse threat landscape, overcoming the scarcity and bias that limit models reliant on existing, often limited, real-world data. This results in a more robust and generalizable model capable of detecting novel and evolving threats. Compared with LLM-based malware detection [21], and in response to RQ4, a key differentiator is our focus on deployability. While [32] demonstrates potential, its reliance on high-quality, structured sandbox data limits real-time applicability. Our model is explicitly designed for resource-constrained edge environments such as industrial IoT systems or smart city infrastructure, ensuring lightweight integration and real-time performance without dependency on complex, centralized behavioral analysis. These design choices enable deployment as a modular AI analysis layer alongside existing NGFWs, operating on commodity edge hardware without GPUs or cloud-based processing.

Our work also builds on studies that combine feature selection with ensemble learning to detect obfuscated malware [31]. Although effective for static analysis accuracy, such approaches often lack NLP integration and do not address real-time or edge scalability constraints. Our model supports interpretability through several mechanisms: Random Forest provides feature-importance rankings that highlight which metadata tokens (e.g., specific ports, protocols, payload snippets) most influence malicious classifications; Logistic Regression yields coefficient weights that indicate linear associations between terms and threat labels; and the soft-voting ensemble outputs class probabilities that convey prediction confidence. These factors further support RQ2 by optimizing accuracy with transparency. At the same time, adoptability is ensured by the model’s lightweight architecture—TF-IDF with a limited vocabulary, fast RF + LR inference (~15 ms per batch), and modular integration that allows it to operate as an optional AI layer within existing NGFW frameworks.

Finally, with respect to RQ4, the latency introduced by the NLP pipeline is the key implementation. While TF-IDF transformation and ensemble inference add computational overhead compared to pure signature matching, our batch-processing approach and reliance on lightweight metadata (rather than full payload analysis) keep the 95th-percentile inference latency under 50 ms for batches of 50 sessions. These characteristics demonstrate that the proposed approach can be deployed as a lightweight, modular AI analysis layer alongside existing NGFWs in constrained edge environments, without requiring specialized hardware, GPUs, or cloud-based processing. This meets the real-time requirements for most edge applications [30]. Future work will explore more efficient feature extraction methods, such as hashing vectorizers, to further minimize this overhead.

We bridge a critical gap between theory and practice identified in foundational reviews [33]. While such reviews lay the theoretical groundwork for NLP in cybersecurity, they often lack applied methodologies and deployment considerations. Our work operationalizes these concepts into a deployable solution, incorporating realistic data balancing and resource profiling to meet the performance demands of real-world NGFW architectures.

### 5.1. Limitations and Future Work

The proposed framework demonstrates strong performance in controlled experiments, but it has several limitations. First, the reliance on TF-IDF may not capture deeper semantic relationships present in natural-language threat reports; future work could explore lightweight transformers (e.g., DistilBERT) with knowledge distillation for edge deployment. Second, our synthetic data generation assumes that SMOTE-ENN produces realistic minority-class samples; adversarial robustness against crafted evasion samples remains an open challenge. Third, the current evaluation uses offline datasets; real-world validation on live edge traffic is needed to assess operational reliability.

Fourth, the entity-relationship graph is currently used only for feature-level augmentation; incorporating lightweight graph neural networks (GNNs) could capture higher-order relational patterns more directly while remaining suitable for edge environments. Finally, the model is designed for text-based logs and metadata; extending it to multimodal inputs (e.g., encrypted traffic fingerprints) could broaden its applicability. While this profiling confirms the model’s inherent lightweight nature, final validation requires implementation on actual edge NGFW hardware (e.g., on a Raspberry Pi running OPNsense). This is planned as immediate future work to obtain definitive latency and power consumption metrics.

### 5.2. Security Considerations and Adversarial Robustness

Deploying ML models in adversarial environments, such as network security, introduces unique risks. An adversary aware of the TF-IDF-based detection could attempt to evade detection by crafting network traffic whose metadata or payload snippets produce TF-IDF vectors that resemble those of benign traffic. Potential attack vectors: (1) Feature manipulation—an attacker could inject benign-looking keywords into malicious payloads or manipulate packet fields to alter the session metadata string. (2) Exploitation of model bias—if the model over-relies on certain “malicious” keywords, an attacker could obfuscate or avoid them.

## 6. Conclusions

This study presents a novel integration of NLP and ensemble learning into next-generation firewalls to enhance malware detection at the edge. By treating log messages and system alerts as analyzable text data, the proposed model uses TF-IDF vectorization and a soft-voting ensemble of classifiers to distinguish between benign and malicious activities. The model demonstrated high accuracy (95% and 98%) across datasets, a strong precision-recall balance, and low misclassification rates. These results confirm that textual indicators often overlooked in traditional systems can be effectively mined to pre-filter or augment threat detection. Consequently, our approach provides a lightweight, explainable, and adaptable framework suitable for deployment in smart cities, industrial IoT, and 5G edge clusters. However, a critical consideration for real-world deployment is the inherent trade-off between high detection accuracy and the computational latency introduced by model inference on resource-constrained devices.

Therefore, future work will focus on overcoming this limitation by optimizing lightweight model variants and leveraging hardware accelerators to minimize inference time without compromising model performance. Finally, real-world generalization will require validation on live traffic logs and multilingual datasets. This study ultimately contributes to a promising AI-driven approach to enhancing firewall intelligence through language-based threat recognition.

## Figures and Tables

**Table 1:** Real-time input data specification for the NLP module.

Data Source	Format Example	Collection Trigger	Processing Latency
Session metadata	src_IP:port_dst_ IP:port_proto_X_ duration_Ys_bytes_out_ Z…”	Session termination OR 30 s window for long sessions	Low (~1 ms per session)
Security alerts	“ALERT: [RuleID] description from [src] to [dst]”	Immediate upon NGFW rule match | Negligible	
Targeted payloads snippets	Hex-encoded string (e.g., ”474554202f2 0485454502f312 e310d0a…”	Anomalous metadata score > threshold	Medium (~5–10 ms per session)
System logs	“Timestamp, Service, Level, Message”	Periodic polling (e.g., every 5 s)	Configurable

**Table 2:** Summary of Simulation Parameters and their Malware.

Parameters	Values
Datasets	Kaggle Cyberthreat Intelligence and CSE-CIC-IDS2018
Text preprocessing for NLP	Tokenization, stop word removal, stemming/lemmatization.
Algorithms	Random Forest, Logistic Regression
Splitting strategy	70% training/validation, 30% testing (with 5-fold CV on training portion for hyperparameter tuning)
Metrics	Accuracy, precision, recall, f1-score, AUC, FPR, FNR, cross-validation
Statistical significance tests	ANOVA and significance level α
Hyperparameter tuning	Method: Bayesian Optimization (50 iterations) - RF: n_estimators [100, 200, 300], max_depth [10, 20, 30, None] - LR: C [0.001, 0.01, 0.1, 1, 10, 100], solver [‘liblinear’,‘lbfgs’] - TF-IDF: max_features [5000, 10,000, 15,000], min_df [1, 2, 3]

**Table 3:** Dataset Descriptive Statistics.

Charateristics	Cyber Threat Dataset	CSE-CIC-IDS2018
Total samples	50,000 (processed/subset used)	10,330,453~16,232,943 (full dataset); ~10,330,453 (our processed subset)
Benign samples	45,000	~13,484,708 (full); 6,067,402 (our subset)
Malicious samples	5000	~2,748,235 (full)
After balancing	75,000 (30,000 synthetic malicious added via SMOTE-ENN)	imbalance handled through stratified sampling, cross-validation, and an ensemble classifier (benign is the dominant class)
Feature dimension	10,000 (TF-IDF)	10,000 (TF-IDF)
Avg.text length	85 tokens	92 tokens
Class imbalance ratio	9:1 (benign:malicious) → ~1.5:1 (45,000 benign: 30,000 malicious)	~1.42:1 (benign: malicious, i.e., benign majority) → effectively mitigated (high performance on both classes)

**Table 4:** Classification report on the cyber threat intelligence dataset (imbalanced setting, TF-IDF + graph features).

Metrics	Traffic Type 0	Traffic Type 1
Precision	0.99	0.22
Recall	1.00	0.07
F1-score	1.00	0.10
Accuracy	0.99	

**Table 5:** Classification Report: Testing and cross-validation, respectively, on a balanced dataset.

Metrics	Class 0	Class 1	Metrics	Class 0	Class 1
Precision	0.9479	0.9462	Precision	0.9509	0.9489
Recall	0.9565	0.9356	Recall	0.9570	0.9417
F1-score	0.9522	0.9408	F1-score	0.9539	0.9453
Accuracy	0.9471		Accuracy		0.9500

**Table 6:** ANOVA for classification performance.

Source of Variation	SS	DF	MS	F-Ratio
Between classes	3.409551	1	3.409551	3.409396
Within classes	44,000	43,998	1.000045	
Total	44,003	43,999		

**Table 7:** Classification report on the CSE-CIC-IDS2018 dataset using the proposed NLP–ensemble model.

Traffic Type	Precision	Recall	F1-Score	Accuracy	Validation
Benign	0.97	0.99	0.98	0.98	0.9779
Attack	0.99	0.97	0.98		

**Table 8:** Performance comparison of the proposed model against baseline classifiers on the balanced cyber threat dataset.

Model	Accuracy	F1-Score	Inference Time	Model Size (mb)
RF	0.938	0.930	12	25
LR	0.925	0.917	3	2
DT	0.910	0.895	2	5
Naïve Bayes	0.901	0.882	3	1
XGBoost	0.945	0.938	22	45
Proposed (RF + LR)	0.950	0.945	15	28

**Table 9:** Comparative analysis of malware detection approaches.

Aspect	NLP–Ensemble Model (Proposed)	LLMs for Behavior-Based Malware Detection [3]	Hybrid Ensemble Learning Combined with Feature Selection [6]	A Review of NLP Techniques for Malware Detection [1]
Technique	Ensemble Model with TF-IDF on synthetically balanced log and metadata.	LLMs for classifying behavioral logs from dynamic analysis	Ensemble learning with multiple feature selection methods	Surveys NLP techniques like NER, topic modeling, and semantic analysis in cybersecurity
Performance metrics	High accuracy (∼0.95, 0.98), Precision, AUC, and ANOVA.	Focuses on detection accuracy and zero-day malware in sandboxes	Evaluates accuracy, precision, recall, and F1-score. Empirical but static.	Purely theoretical, no empirical benchmarks or implementation.
Novelty	Synthetic data balancing for imbalanced threats; edge-deployable, text-driven detection pipeline.	Applying large-scale LLMs to malware behavior analysis	Combining feature selection with ensemble models for obsfuscated malware	Synthesizes how NLP methods apply to malware detection
Application	Real-time detection in NGFWs, SOC tools, and edge clusters (IoT, Smart cities).	Post hoc, sandbox-based dynamic malware analysis	Non-real-time detection of obfuscated malware	Conceptual guidance for research and system design
Limitations	Language variability, no packet-level payload inspection	High computational cost, not suitable for real-time or edge deployment	Lacks NLP and real-time capabilities; limited to static analysis	No implementation or performance analysis, nor a deployable solution

## Data Availability

The datasets used in this study are publicly available. The cyber threat intelligence and CSE-CIC-IDS2018 can be accessed on Kaggle. These datasets were utilized to conduct the analysis presented in this study.

## Abbreviations

The following abbreviations are used in this manuscript:

ANOVA	Analysis of Variance
AI	Artificial Intelligence
AUC	Area Under the Curve
FRP	False Rate Positive
FNR	False Negative Rate
GNN	Graph Neural Network
IP	Internet Protocol
IOCs	Indicators of Compromises
IIoTs	Industrial Internet of Things
LR	Logistic Regression
LLMs	Large Language Models
ML	Machine Learning
NLP	Natural Language Processing
NGFWs	Next-Generation Firewalls
RF	Random Forest
SOC	Security Operations Centre
TF-IDF	Term Frequency-Inverse Document Frequency
C2	Command and control
SMOTE-ENN	Synthetic Minority Oversampling Technique with Edited Nearest Neighbors (SMOTE-ENN)
CV	Cross-validation
IoT	Internet of Things
5G	Fifth generation mobile network
NER	Name Entity Recognition
